# High serum glial fibrillary acidic protein levels are associated with increased risk of post-stroke cognitive impairment

**DOI:** 10.3389/fnagi.2025.1546270

**Published:** 2025-05-12

**Authors:** Wanying Shan, Rui Jiang, Jing Wang, Guoli Xu, Jie Zhao, Guojie Zhai, Jiaxin Shao

**Affiliations:** ^1^Department of Neurology, Suzhou Ninth People’s Hospital, Soochow University, Suzhou, China; ^2^Department of Gerontology, Suzhou Ninth People’s Hospital, Soochow University, Suzhou, China

**Keywords:** acute ischemic stroke, cognitive impairment, GFAP, biomarker, prediction

## Abstract

**Background and purpose:**

The correlation between glial fibrillary acidic protein (GFAP) and cognitive impairment in acute ischemic stroke patients remains uncertain. We aimed to evaluate GFAP in serum as predictor of post-stroke cognitive impairment (PSCI) at 90 days.

**Methods:**

From March 2022 to February 2023, patients with first-ever ischemic stroke were prospectively enrolled. Serum GFAP concentrations were measured within 24 h after admission using an enzyme-linked immunosorbent assay. Cognitive function measurement was performed at the 90 days follow-up using the Mini-mental state examination (MMSE). A MMSE score <27 was defined as PSCI. Multiple logistic regression and restricted cubic spline were performed to examine the association between GFAP and cognitive impairment.

**Results:**

A total of 336 patients (mean age: 66.3 ± 9.0 years; 58.3% male) with acute ischemic stroke were included. The median GFAP levels were 0.73 ng/mL (interquartile range, 0.38–1.09 ng/mL). During the 3-month follow-up, 164 participants (48.8%) experienced PSCI. Higher GFAP levels were independently associated with PSCI after adjusting for potential confounders (per 1-unit increase, odds ratio: 3.91; 95% confidence interval: 2.24–6.82; *p* = 0.001). Additionally, restricted cubic spline confirmed a linear relationship between serum GFAP concentrations and PSCI risk (*P* for linearity = 0.001).

**Conclusion:**

This study demonstrated that higher levels of GFAP were associated with PSCI, suggesting that GFAP could be a promising and straightforward screening indicator of cognitive impairment after stroke.

## 1 Introduction

Stroke is one of the leading causes of death and disability in China (Wang et al., 2022). Post-stroke cognitive impairment (PSCI) is a common complication after ischemic stroke, with the prevalence ranging from 20 to 80% for cognitive impairment, and up to 30% for dementia within the first year after stroke onset (Brainin et al., 2015; Sun et al., 2014; Yang et al., 2024; Barbay et al., 2009). Previous studies have consistently demonstrated that PSCI may affect the physical recovery and quality of life and is related to an elevated risk of stroke recurrence, death and functional impairment (Gottesman and Hillis, 2010; Oksala et al., 2009; Melkas et al., 2009). Therefore, finding effective predictive factors and implementing patient risk stratification management of PSCI is of great clinical value. One hypothesis is that ischemic stroke may trigger a degenerative process that causes cognitive dysfunction and is reported to involve neuroinflammation (Thiel et al., 2014).

GFAP is a brain-specific intermediate filament protein primarily expressing in astrocytes and is secreted from damaged or activated astrocytes (Eng et al., 2000; Ribotta et al., 2004). Growing evidence supports the potential clinical use of blood GFAP levels in numerous neuroinflammatory and neurodegenerative diseases including head trauma, multiple sclerosis, intracerebral hemorrhage, and ischemic stroke (Vos et al., 2004; Foerch et al., 2012; Kraljević et al., 2024; Ayrignac et al., 2020). In our literature, we found that elevated GFAP concentrations were related to the presence of depression after ischemic stroke, which indicated that Neuroinflammatory response may be involved in post-stroke complications (Shan et al., 2023). Nevertheless, the relationship between GFAP and PSCI in acute ischemic stroke patients is not clear yet. Moreover, GFAP displayed predictive value for risk of Alzheimer disease progression and cognitive decline (Shen et al., 2023).

The present study aimed to explore the association between serum GFAP levels and PSCI, which may aid to identify potential biomarkers for the detection and prevention of PSCI.

## 2 Methods

### 2.1 Study participants

This prospective study recruited consecutive patients with first-ever ischemic stroke admitted in Suzhou Ninth People’s Hospital, between March 2022 and February 2023. Patients were enrolled if they fulfilled the following inclusion criteria: (1) aged 18 years or old; (2) ischemic stroke was diagnosed according to the criterion of World Health Organization (Stroke, 1989); (3) time lapse between the symptom onset and hospitalization < 7 days. Patients were excluded from this study if they: (1) had pre-stroke diagnosis of any psychiatric illness, major depressive disorder, cerebral trauma, Alzheimer’s Disease, Parkinson’s disease, alcoholic abuse, central nervous system disease, renal failure, hepatic failure, autoimmune disease and thyroid hormone disorders; (2) had severe neurological deficit that precluded us from performing the psychological measurement. Ethical approval was given by the local ethical committee of Suzhou Ninth People’s Hospital. Written informed consent was obtained directly from all participants or, in cases of impaired decision-making capacity, from their legally authorized representative.

### 2.2 Data collection

Data was obtained using a standard questionnaire at admission, which includes demographic characteristics, clinical data, medical and medication history, and ischemic stroke subtype. Baseline stroke severity was assessed by trained neurologists using the National Institutes of Health Stroke Scale (NIHSS) (Brott et al., 1989). Infarction volume was assessed by the DWI based Alberta Stroke Program Early CT Score (DWI-ASPECTS). Ischemic stroke etiology was defined using the Trial of Org 10,172 in Acute Stroke Treatment (TOAST) classification (Adams et al., 1993). Laboratory data including lipid profile, glucose levels and hyper-sensitive C-reactive protein were also recorded.

### 2.3 Biomarker measurement

Fasting venous blood samples were drawn within 24 h after admission from all the patients, which centrifuged at 1500 × g for 15 min (4°C) to obtain serum. Serum was separated immediately and frozen at −80°C until a predetermined number of samples was obtained. Before examining serum GFAP levels, the instrument will calibrate. Commercially available enzyme-linked immunosorbent assay (ELISA) kit was used to measure the GFAP levels. The lower limit of quantification for GFAP was established at 0.1 ng/mL. The coefficients of intra-assay variability and inter-assay for GFAP were found to be 10.0 and 15.0%, respectively. To assess assay precision, a randomly selected subset of 10% study samples underwent duplicate analysis under identical processing conditions. All laboratory procedures were conducted by certified technicians who were blinded to the baseline characteristics and clinical outcomes.

### 2.4 Outcome assessment

The cognitive assessments were conducted at 3 months (+/− 1 week) after the acute ischemic stroke by trained neurologists (G.X. and J.Z.) who were blinded to the patients’ baseline data. Cognitive function was measured using the Mini-mental State Examination (MMSE). The MMSE has been confirmed to be a valid screening tool for assessing cognitive impairment and dementia in the Chinese population. According to previous literatures (Delavaran et al., 2017; Webb et al., 2014; Pendlebury et al., 2010), PSCI was defined as a MMSE score < 27. Furthermore, PSCI severity was categorized as follows: severe cognitive impairment (MMSE score, 0–22), mild cognitive impairment (MMSE score, 23–26), and no cognitive impairment (MMSE score, 27–30).

### 2.5 Statistical analysis

All analyses were conducted using SPSS for Windows, version 24.0 (SPSS Inc., Chicago, IL, USA) and R statistical software version 4.0.0 (R Foundation, Vienna, Austria). Continuous variables were presented as the mean ± standard deviation or median (interquartile range) and categorical variables were presented as numbers (percentages). Categorical variables were compared by the Pearson’s chi-square test or Fisher exact test, and continuous variables were compared by the *t*-test, Mann–Whitney U test, one-way analysis of variance, and Kruskal-Wallis test. Binary logistical regression analysis was utilized to assess the correlation between serum GFAP levels and PSCI. The effect of GFAP levels on PSCI severity was assessed using ordinal logistic regression analysis. Variables with *p* < 0.1 in the univariate analysis were entered into the backward selection model. Those demonstrating *p* < 0.05 in the likelihood ratio test were retained in the final model. Missing data in key variables (baseline NIHSS score and DWI-ASPECTS) were handled through multivariate imputation using chained equations with five imputed datasets, and the variables were missing at random. We further performed the variance inflation factor (VIF) to evaluate multicollinearity in regression model. A VIF > 10 as an indicator of multicollinearity. Spline regression model was used to achieve a more accurate evaluation of the shape of the association between circulating GFAP concentrations and cognitive impairment, fitting a restricted cubic spline function with 3 knots (at the 5th, 55th, and 95th percentiles) (Durrleman and Simon, 1989). Additionally, a receiver operating characteristic curve was constructed to assess the discriminatory power of risk factors in predicting PSCI. A 2-tailed *p* < 0.05 was considered to be statistically significant.

In this study, sample size calculation was performed using MedCalc software, based on preliminary data from 50 PSCI patients and 50 non-PSCI controls, where serum GFAP levels demonstrated a mean intergroup difference of 0.15 ng/mL. With *α*-level set at 0.05 (two-tailed) and statistical power of 80%, the calculated required sample size was 312 participants. To account for potential 5% attrition rate in 90-day cognitive function follow-up, the final sample size was adjusted upward to 330 participants.

## 3 Results

### 3.1 Baseline characteristics

**Figure 1:**
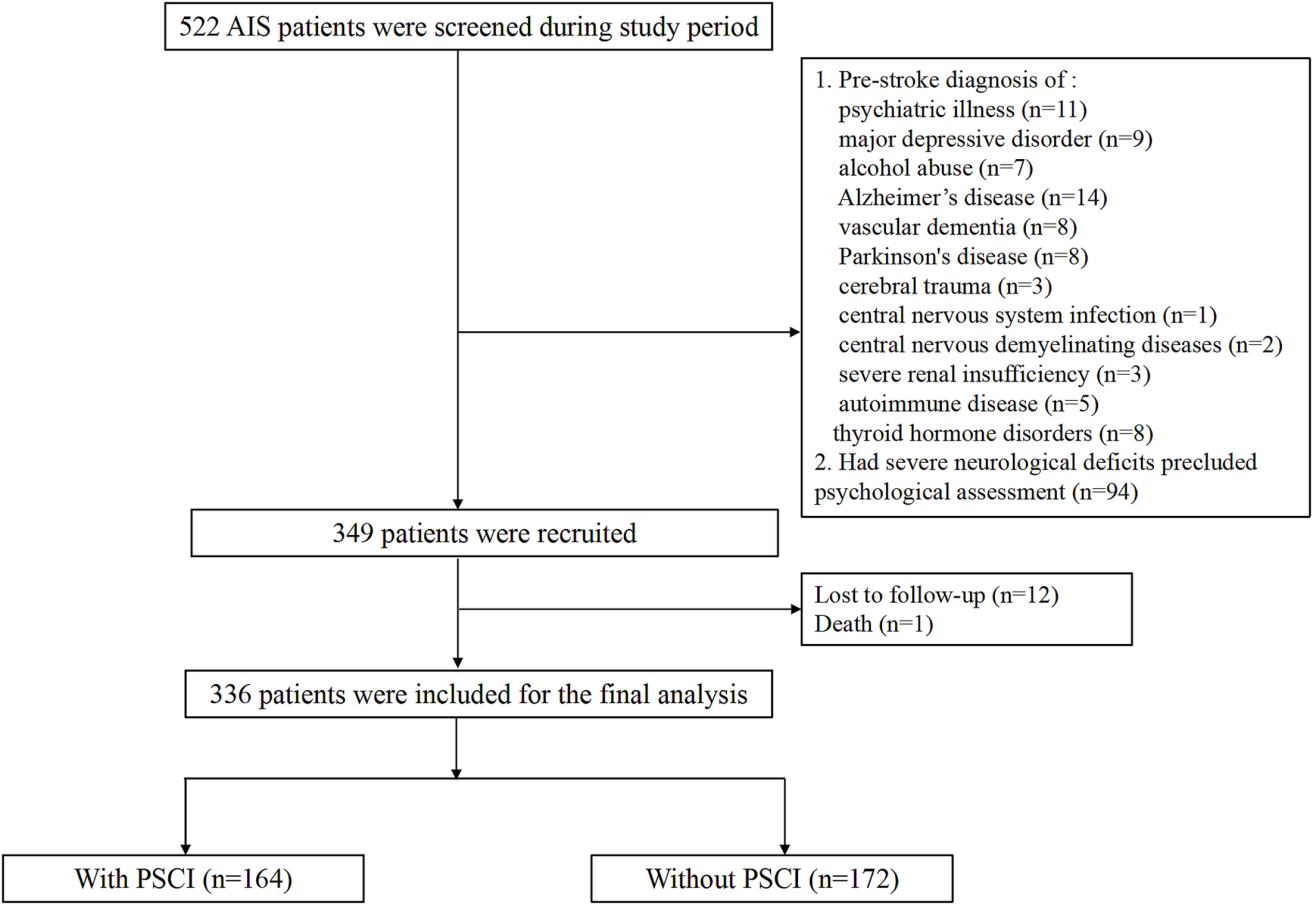
Flow chart of participants’ selection.

A total of 336 ischemic stroke patients (median age 66.3 years; 56.8% male) were finally recruited. The flow diagram was demonstrated in Figure 1. Vascular risk factors were distributed as follows among the 336 enrolled patients: 232 cases (69.0%) of hypertension, 87 (25.9%) with diabetes mellitus, and 52 (15.5%) exhibiting hyperlipidemia. WMLs was found in 148 (44.0%) patients. The median NIHSS score at admission was 5.0 points. The median circulating GFAP concentration was 0.73 ng/mL (interquartile range, 0.38–1.09 ng/mL).

### 3.2 Factors associated with PSCI

**Figure 2:**
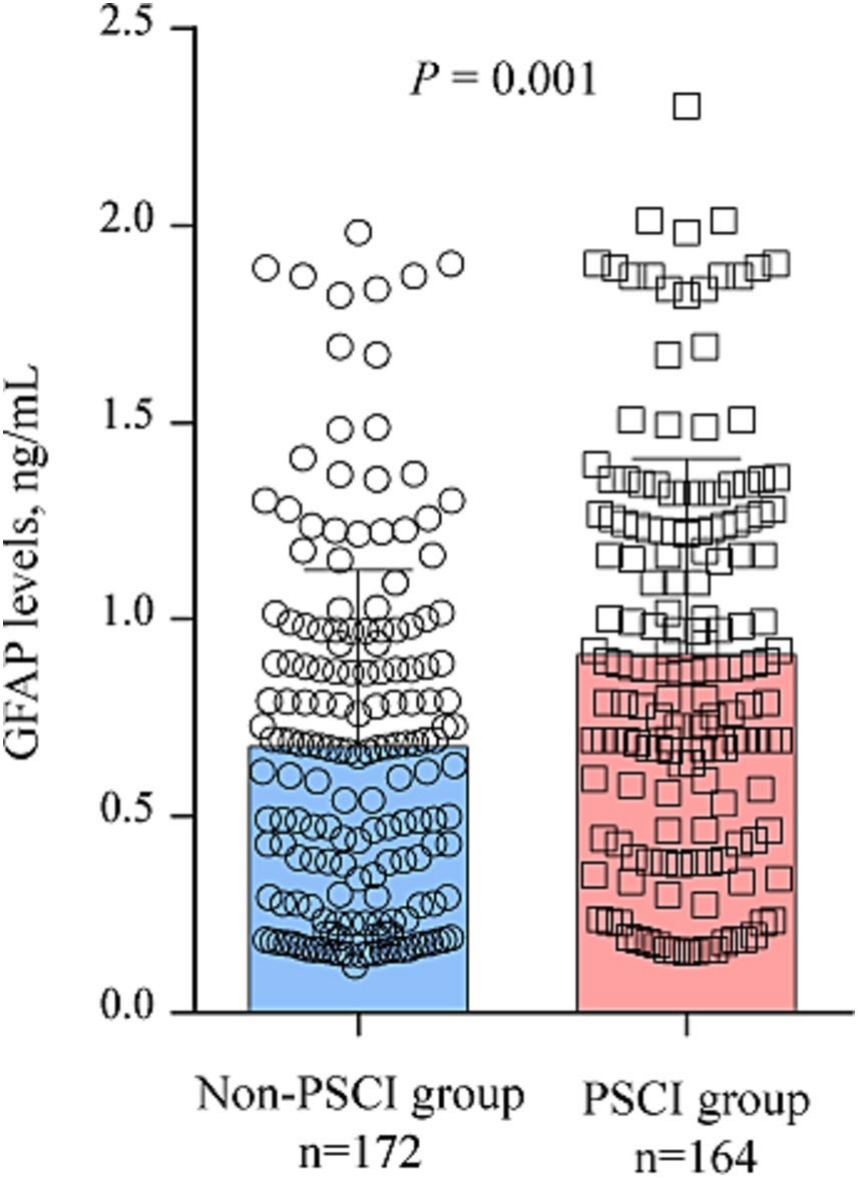
Distribution of the GFAP levels according to patients with and without PSCI.

According to MMSE score at 3 months, 164 participants (48.8%) had PSCI The comparison of clinical data between patients with and without PSCI were demonstrated in Table 1. Those with PSCI tended to be older (*p* = 0.003), had higher prevalence of hypertension (*p* = 0.039), diabetes (*p* = 0.001), DWI-ASPECTS 0–7 (*p* = 0.041) and WMLs (*p* = 0.012), and have higher NIHSS score (*p* = 0.041), and hyper-sensitive C-reactive protein levels (*p* = 0.001). Figure 2 showed the distribution of GFAP levels stratified by the status of cognitive function. GFAP levels were higher in patients with PSCI than in patients without (median 0.87 ng/mL versus 0.66 ng/mL; *p* = 0.001).

**Table 1:** Results of the comparisons between patients with and without PSCI.

Variables	With PSCI, *n* = 164	Without PSCI, *n* = 172	*P*-value
Demographic characteristics			
Age, year	67.9 ± 8.2	64.9 ± 9.6	0.003
Male, %	89 (54.3)	102 (59.3)	0.352
Vascular risk factors, *n* (%)			
Hypertension	122 (74.4)	110 (64.0)	0.039
Diabetes mellitus	60 (36.6)	27 (15.7)	0.001
Hyperlipidemia	22 (13.4)	30 (17.4)	0.308
Coronary heart disease	23 (14.0)	22 (12.8)	0.740
Current smoking	58 (35.4)	68 (39.5)	0.430
Clinical data			
Systolic blood pressure, mmHg	142.5 ± 16.0	139.3 ± 14.7	0.191
Diastolic blood pressure, mmHg	81.8 ± 10.6	80.1 ± 10.4	0.134
Baseline NIHSS, score*	5.5 (3.0, 8.0)	5.0 (2.0, 8.0)	0.041
DWI-ASPECTS 0–7, *n* (%)^#^	83 (50.6)	65 (37.8)	0.041
White matter lesions, *n* (%)	84 (51.2)	64 (37.2)	0.012
Stroke subtypes, *n* (%)			0.673
Large artery atherosclerosis	68 (41.5)	81 (47.1)	
Cardioembolism	32 (19.5)	33 (19.2)	
Small vessel occlusion	48 (29.3)	41 (23.8)	
Others	16 (9.8)	17 (9.9)	
Laboratory data			
Total cholesterol, mmol/L	4.2 ± 1.2	4.1 ± 1.1	0.433
Triglyceride, mmol/L	1.4 (1.0, 1.8)	1.3 (1.1, 1.7)	0.507
Low density lipoprotein, mmol/L	2.3 (1.9, 2.8)	2.3 (2.0, 3.1)	0.107
High density lipoprotein, mmol/L	1.1 ± 0.2	1.1 ± 0.3	0.679
Baseline glucose levels, mmol/L	6.0 ± 2.4	5.7 ± 2.4	0.145
Hyper-sensitive C-reactive protein, mg/L	7.8 (3.3, 11.0)	4.5 (2.2, 9.4)	0.001
GFAP, ng/mL	0.87 (0.56, 1.24)	0.66 (0.27, 0.94)	0.001

NIHSS, national institutes of health stroke scale; GFAP, glial fibrillary acidic protein; PSCI, post-stroke cognitive impairment. *13 patients missed the baseline NIHSS score; ^#^28 patients missed the DWI-ASPECT score.

Table 2 illustrated the results of binary regression analyses for the association of risk factors with PSCI. The univariate regression analysis demonstrated that age [odd ratio (OR) 1.04, 95% confidence interval (CI), 1.01–1.07, *p* = 0.003], hypertension (OR 1.64, 95% CI, 1.02–2.62, *p* = 0.039), diabetes mellitus (OR 3.10, 95% CI, 1.84–5.21, *p* = 0.001), baseline NIHSS score (OR 1.08, 95% CI, 1.02–1.14, *p* = 0.014), DWI-ASPECTS 0–7 (OR 1.60, 95% CI, 1.02–2.51, *p* = 0.041), WMLs (OR 1.77, 95% CI, 1.15–2.74, *p* = 0.010), hyper-sensitive C-reactive protein levels (OR 1.05, 95% CI, 1.01–1.09, *p* = 0.017) and GFAP levels (per 1-unit increase, OR 2.84, 95% CI, 1.76–4.57, *p* = 0.001) were significantly associated with PSCI.

**Table 2:** Binary regression analyses for the association of risk factors with PSCI.

Variables	Univariate regression analysis		Multivariate regression analysis*	
	OR (95%CI)	*P*-value	OR (95%CI)	*P*-value
Age	1.04 (1.01–1.07)	0.003	1.03 (1.00–1.06)	0.035
Male	0.80 (0.53–1.26)	0.352	–	–
Hypertension	1.64 (1.02–2.62)	0.039	–	–
Diabetes mellitus	3.10 (1.84–5.21)	0.001	3.71 (2.02–6.84)	0.001
Hyperlipidemia	0.73 (0.40–1.33)	0.309		
Coronary heart disease	1.11 (0.59–2.08)	0.740		
Current smoking	0.84 (0.54–1.30)	0.430		
Systolic blood pressure	1.01 (0.99–1.02)	0.191		
Diastolic blood pressure	1.02 (0.99–1.04)	0.135		
Baseline NIHSS	1.08 (1.02–1.14)	0.014	–	–
DWI-ASPECTS 0–7	1.60 (1.02–2.51)	0.041	–	–
White matter lesions	1.77 (1.15–2.74)	0.010	–	–
Stroke subtypes				
Large artery atherosclerosis	0.89 (0.42–1.90)	0.767		
Cardioembolism	1.03 (0.45–2.38)	0.994		
Small vessel occlusion	1.24 (0.60–2.77)	0.593		
Others	Reference			
Laboratory data				
Total cholesterol	1.71 (0.89–1.30)	0.432		
Triglyceride	1.041 (0.77–1.41)	0.781		
Low density lipoprotein	0.77 (0.59–1.09)	0.079	–	–
High density lipoprotein	1.22 (0.49–3.04)	0.678		
Baseline glucose levels	1.70 (0.98–1.17)	0.148		
Hyper-sensitive C-reactive protein	1.05 (1.01–1.09)	0.017	–	–
GFAP levels	2.84 (1.76–4.57)	0.001	3.91 (2.24–6.82)	0.001

CL, Confidence interval; GFAP, glial fibrillary acidic protein; OR, Odd ratio; PSCI, post-stroke cognitive impairment. *The multivariate model was adjusted for demographic characteristics and *P* < 0.1 in the univariate analysis.

**Figure 3:**
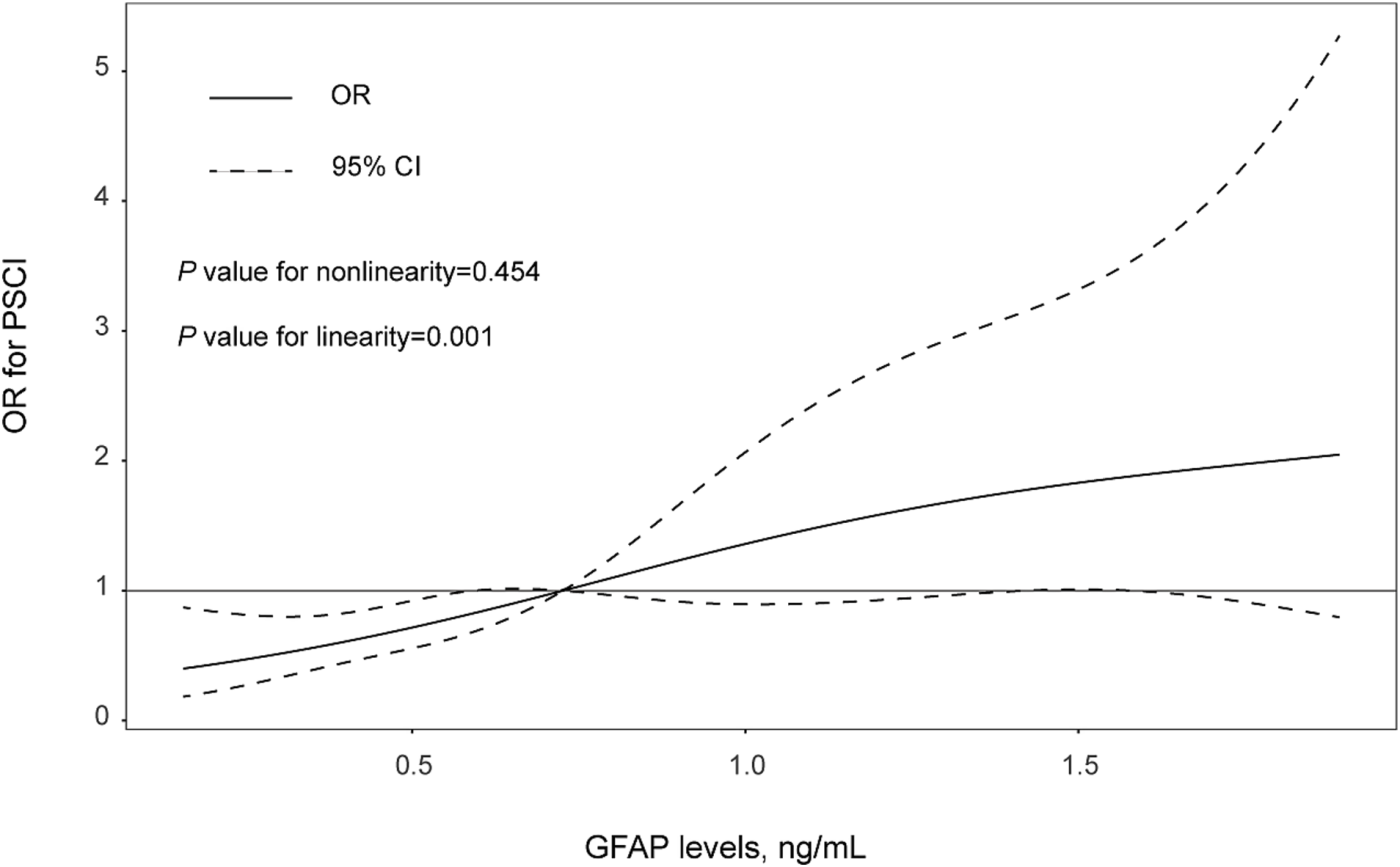
The association between levels of GFAP levels and PSCI risk were evaluated with restricted cubic spline. Solid lines are adjusted odds ratios, with daseed lines showing 95% confidence intervals derived from restricted cubic spline regressions with three knots (placed at the 5th, 50th and 95th percentiles of GFAP levels). Reference lines for no association are indicated by the solid gray lines at an odd ratio of 1.0. Analysis was adjusted for age, sex, hypertension, diabetes mellitus, baseline NIHSS score, WMLs, and hyper-sensitive C-reactive protein levels.

There was not any multicollinearity of GFAP levels with age, hypertension, diabetes, baseline NIHSS score, DWI-ASPECTS 0–7, WMLs and hyper-sensitive C-reactive protein levels (all VIF < 10). The multivariate model, adjusted for demographic characteristics and *p* < 0.1 in the univariate analysis, identified 3 significant and independent predictors of PSCI: advancing age (OR 1.03, 95% CI, 1.00–1.06, *p* = 0.035), pre-existing diabetes (OR 3.71, 95% CI, 2.02–6.84, *p* = 0.001), and elevated GFAP concentrations (per 1-unit increase, OR 3.906, 95% CI, 2.24–6.82, *p* = 0.001). Furthermore, multiple-adjusted spline regression analysis indicated a positive dose–response association of serum GFAP with PSCI risk at 3 months (*p-*value for linearity = 0.001; Figure 3).

**Figure 4:**
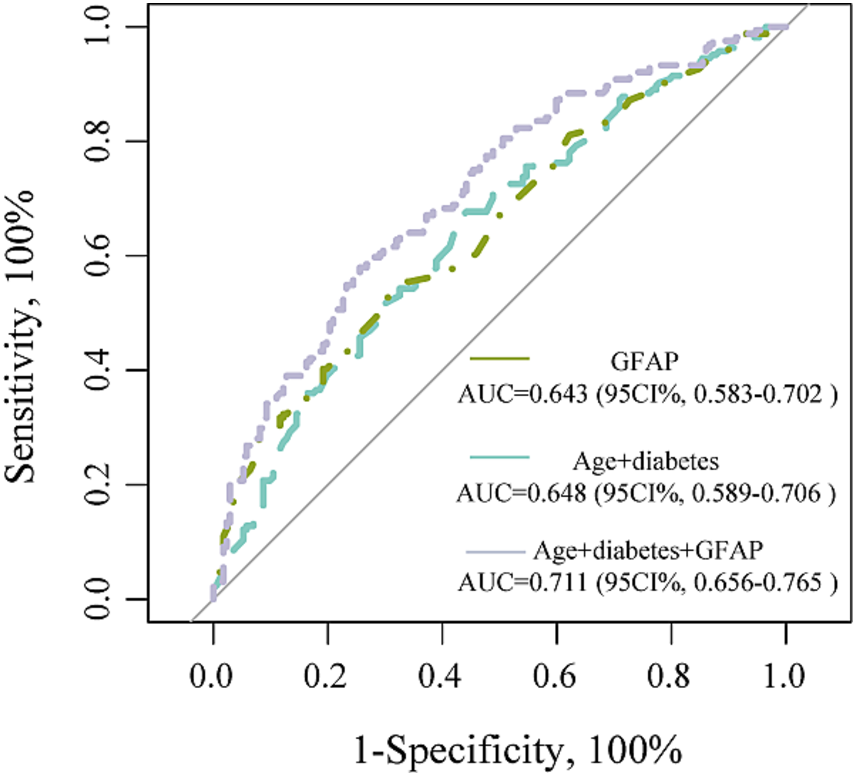
ROC curve in predicting PSCI.

We further performed the ROC curve to detect the discriminatory ability of different models in predicting PSCI. The AUC results were analyzed using data from all patients. The incorporation of GFAP into the traditional risk factor model significantly improved the predictive performance for PSCI, as evidenced by an increase in the area under the receiver from 0.648 to 0.711 (*p* = 0.012; Figure 4).

### 3.3 Association between GFAP levels and the severity of PSCI

Among these PSCI patients, 64 (39.0%) patients had mild PSCI, and 100 (61.0%) patients had severe PSCI. Multivariate ordinal regression analysis further confirmed that higher serum GFAP levels were significant associated with PSCI severity (per 1-unit increase, OR 5.02, 95% CI, 2.95–8.55, *p* = 0.001; Table 3).

**Table 3:** Ordinal regression analyses for the association of risk factors with PSCI severity.

Variables	Univariate regression analysis		Multivariate regression analysis*	
	OR (95%CI)	*P*-value	OR (95%CI)	*P*-value
Age	1.03 (1.09–1.06)	0.008	–	–
Male	1.13 (0.75–1.71)	0.547	–	–
Hypertension	0.64 (0.43–1.06)	0.083		
Diabetes mellitus	2.10 (1.32–3.33)	0.002	2.34 (1.34–4.08)	0.003
Hyperlipidemia	1.46 (0.82–2.60)	0.204		
Coronary heart disease	0.91 (0.50–1.65)	0.757		
Current smoking	1.16 (0.76–1.77)	0.493		
Systolic blood pressure	1.01 (0.99–1.02)	0.226		
Diastolic blood pressure	1.02 (1.00–1.04)	0.044		
Baseline NIHSS	1.09 (1.04–1.21)	0.001	1.08 (1.01–1.15)	0.026
DWI-ASPECTS 0–7	1.59 (1.03–2.42)	0.034	–	–
White matter lesions	2.03 (1.34–3.07)	0.001	–	–
Stroke subtypes				
Large artery atherosclerosis	0.93 (0.45–1.91)	0.848		
Cardioembolism	1.11 (0.52–2.47)	0.791		
Small vessel occlusion	1.25 (0.37–1.71)	0.559		
Others	Reference			
Laboratory data				
Total cholesterol	1.07 (0.90–1.28)	0.458		
Triglyceride	1.07 (0.81–1.43)	0.630		
Low density lipoprotein	1.30 (0.99–1.67)	0.068	-	-
High density lipoprotein	1.26 (0.54–3.00)	0.603		
Baseline glucose levels	1.06 (0.98–3.14)	0.155		
Hyper-sensitive C-reactive protein	1.03 (0.99–1.07)	0.056	-	-
GFAP levels	4.00 (2.54–6.30)	0.001	5.022 (2.95–8.55)	0.001

CL, Confidence interval; GFAP, glial fibrillary acidic protein; OR, Odd ratio; PSCI, post-stroke cognitive impairment. *The multivariate model was adjusted for demographic characteristics and *P* < 0.1 in the univariate analysis.

## 4 Discussion

The present prospective study is the first to explore the correlation between serum GFAP concentrations and PSCI in a Chinese population. We found that GFAP levels were positively and independently associated with PSCI among ischemic stroke patients after adjustment for several established confounders including demographic characteristics, hypertension, diabetes, baseline NIHSS score, DWI-ASPECT, white matter lesions and Hs-CRP levels. Our study provided evidence supporting that serum GFAP may have an important role in the prediction and treatment of cognitive impairment after stroke.

GFAP is a monomeric intermediate filament protein found in the astroglial cytoskeleton, and its release occurs solely after brain injury (Chmielewska et al., 2018). A number of literatures have investigated the role of GFAP as a prognostic marker of functional outcome after acute cerebrovascular accidents (Pujol-Calderón et al., 2022; Liu and Geng, 2018; Li et al., 2024). In a single-center prospective study with 286 ischemic stroke patients (Liu and Geng, 2018), multivariate analysis showed that increased GFAP concentrations at admission independently predicted unfavorable outcome during the 1-year follow-up. Another recent-published study with 142 large vascular occlusive stroke patients confirmed a significant correlation between serum GFAP levels and symptomatic intracranial hemorrhage after endovascular treatment (Li et al., 2024). Similar to ischemic stroke, plasma concentrations of GFAP concentration at baseline were significantly associated with poor outcomes after subarachnoid hemorrhage, as observed by Zheng et al. (2017) at 6 months after the symptom onset (Zheng et al., 2017). Furthermore, after adjusting or combining with other markers, higher levels of plasma GFAP displayed predictive value for risk of the development of Alzheimer’s disease and cognitive decline (Shen et al., 2023; Parvizi et al., 2022). Our study built upon the prior discoveries by suggesting that circulating GFAP concentration could also serve as a promising predictor of cognitive dysfunction after ischemic stroke, which may help clinicians better understand the pathology of PSCI and further guide health care professionals in implementing targeted monitoring and personalized interventions for individuals identified as high risk.

The mechanisms underlying the inverse association of serum GFAP levels with cognitive impairment after ischemic stroke are unclear, but several potential pathways have been proposed. First, the elevated of GFAP might reflect ongoing neuronal degeneration and glial activation due to poststroke immune and inflammatory processes (Heimfarth et al., 2022). Neuroinflammation is an important factor of cognitive decline in the ischemic stroke patients (Thiel et al., 2014). Second, impaired induction of blood–brain barrier properties in aortic endothelial cells was found in *GFAP*^−/−^ mice (Pekny et al., 1998) Blood–brain barrier dysfunction appears early in cerebral hypoperfusion, leading to the deterioration of white matter and cognitive impairment (Xu et al., 2022). Interestingly, our data also found that higher levels of GFAP were associated with an increased risk of withe matter lesions. Third, GFAP levels were correlated with size of infarction. This prospective study observed consistent results that serum GFAP levels positively associated with the infarct volume and the severity of neurological deficits (Pujol-Calderón et al., 2022). In the experimental models of focal cerebral ischemia, Li et al. (2017) generated a GFAP promoter-controlled IL-15–expressing transgenic mouse line, and found enlarged brain infarcts and exacerbated neurological deficits (Li et al., 2017). These results suggested that GFAP may be involved in PSCI by mediating the infarct volume progression after stroke. Further studies are warranted to elucidate these potential mechanisms.

However, our study has several limitations should be addressed. Firstly, it was difficult for us to obtain a clear causal correlation between GFAP and the cognitive impairment due to the cross-sectional study design. Secondly, patients with severe neurological deficits and aphasia could not fully cooperate with scale evaluation. We therefore only performed cognitive assessment in patients with mild to moderate stroke, which may induce the underestimate of real incidence of PSCI. Thirdly, our study focused on 90-day cognitive outcomes and did not include acute-phase cognitive assessments. This design limits our ability to disentangle the potential confounding influence of pre-existing cognitive deficits from true post-stroke cognitive decline. Finally, we detected the GFAP concentrations only at baseline; therefore, we were unable to examine the association between dynamic changes in GAFP and PSCI. In addition, the moderate discriminative capacity of GFAP alone, which may reflect cohort heterogeneity, sample size constraints, or assay sensitivity thresholds. However, while statistical significance does not equate to clinical relevance, our findings still provide a rationale for exploring GFAP’s role in specific subpopulations (e.g., severe disease subtypes or defined pathological stages) where its utility may be amplified. Further dynamic studies are warrant to evaluate these relationships.

In summary, our data demonstrated that higher serum levels of GFAP are associated with increased risk of cognitive impairment measured at 90-days after stroke. Further experimental and cohort studies are warrant to validate our findings and clarify the potential mechanisms.

## Data Availability

The raw data supporting the conclusions of this article will be made available by the authors, without undue reservation.
